# The Biolog EcoPlate™ Technique for Assessing the Effect of Metal Oxide Nanoparticles on Freshwater Microbial Communities

**DOI:** 10.3390/nano11071777

**Published:** 2021-07-08

**Authors:** Imre Németh, Szabina Molnár, Emese Vaszita, Mónika Molnár

**Affiliations:** Department of Applied Biotechnology and Food Science, Faculty of Chemical Technology and Biotechnology, Budapest University of Technology and Economics, H-1111 Budapest, Hungary; nemeth.imre@vbk.bme.hu (I.N.); szabina.molnar@edu.bme.hu (S.M.); vaszita.emese@vbk.bme.hu (E.V.)

**Keywords:** Biolog EcoPlate™, freshwater microbial community, biodiversity, metal oxide nanoparticles, nano titanium dioxide, nano zinc oxide

## Abstract

The application of Biolog EcoPlate™ for community-level physiological profiling of soils is well documented; however, the functional diversity of aquatic bacterial communities has been hardly studied. The objective of this study was to investigate the applicability of the Biolog EcoPlate™ technique and evaluate comparatively the applied endpoints, for the characterisation of the effects of metal oxide nanoparticles (MONPs) on freshwater microbial communities. Microcosm experiments were run to assess the effect of nano ZnO and nano TiO_2_ in freshwater at 0.8–100 mg/L concentration range. The average well colour development, substrate average well colour development, substrate richness, Shannon index and evenness, Simpson index, McIntosh index and Gini coefficient were determined to quantify the metabolic capabilities and functional diversity. Comprehensive analysis of the experimental data demonstrated that short-term exposure to TiO_2_ and ZnO NPs affected the metabolic activity at different extent and through different mechanisms of action. TiO_2_ NPs displayed lower impact on the metabolic profile showing up to 30% inhibition. However, the inhibitory effect of ZnO NPs reached 99% with clearly concentration-dependent responses. This study demonstrated that the McIntosh and Gini coefficients were well applicable and sensitive diversity indices. The parallel use of general metabolic capabilities and functional diversity indices may improve the output information of the ecological studies on microbial communities.

## 1. Introduction

Microbial diversity in aquatic and terrestrial environments is a key component of ecosystem functioning, consequently contamination of these environmental matrices poses potential threats to the structure and function of the respective ecosystems [1,2,3]. Nano titanium dioxide (nTiO_2_) and nano zinc oxide (nZnO) belong to the anthropogenic engineered metal oxide nanoparticles (MO-ENPs), the production and use of which was estimated to increase exponentially during the years owing to their extensive application in medicine, electronics, cosmetic and textile industry [4,5], but recently also in modern agriculture [6]. The likeliness of nTiO_2_ and nZnO release into the environment is higher than that of other ENPs, given their high global production and consumption volume among the MONPs [7] and the several transport pathways. Some studies have reported the presence of anthropogenic ENPs, particularly TiO_2_, in streams [8], wastewater treatment plants [9,10] and lakes [11]. Once released into the aquatic environment, MONPs have been reported to be involved in homo- and hetero-aggregation followed by precipitation or sedimentation and accumulation [12,13,14,15].

Furthermore, in the presence of ligands (Cl^–^, SO_4_^2–^, NO^3–^ etc.), ENPs may form complexes and adsorb onto natural organic matter (NOM) [15]. Therefore, the stability of ENPs in the aquatic ecosystems is influenced by environmental factors and the properties of NPs [16], which in turn will influence their fate and effect on aquatic biota.

On the other hand, the effect of MONPs in the environment is different to their bulk size or dissolved ion counterparts, because of their high surface-to-volume ratio and surface characteristics, such as the intensified charge and reactivity [17]. In addition, the ecotoxicity of MONPs is also determined by further factors including size, oxidative state, exposure time, particle concentration and the target organism and its habitat [18,19,20,21]. As reviewed by Parada et al. [22], there are numerous nano-ecotoxicological studies on the potential toxicity of MONPs involving single species aquatic model organisms, such as daphnids, zebrafish, algae, freshwater bivalve, diatom.

The significance of microbial activity concerning the ecosystem functionality and services is well established, but microbial diversity of natural waters—especially the effect of NPs on the biodiversity and structure of the complex aquatic microbial community—has not been studied extensively. There are some valuable results on this topic based on various approaches focused on rivers [15,23], a stream [13], marine estuarine sediments [24], groundwater [25], and three Swedish lakes [11], nevertheless, the effect of nTiO_2_ and nZnO on complex aquatic microbial communities needs further investigations and novel approaches, and Biolog EcoPlate™ could be a great tool for this aim.

Battin et al. [13] provided the first evidence that nano-TiO_2_ significantly impacted natural microbial communities (bacteria stream biofilms) under natural levels of UV radiation and TiO_2_ concentrations expected in surface waters. To examine the effect of nano-TiO_2_ on the membrane integrity of planktonic microorganisms and biofilm cells in stream water microcosms, nucleic acid stains and epifluorescence microscopy was used [13].

Farkas et al. [11] conducted the first ecotoxicological study assessing the effect of nTiO_2_ on bacterial communities *in situ*, in three lakes, taking into consideration dissolved organic carbon (DOC) concentrations, element concentrations and light regimes. Bacterial growth after the termination of the exposure period was determined as abundance in the water samples analysed by flow cytometry. Their results demonstrated that TiO_2_ NPs could affect natural lake-water bacterial communities in terms of bacterial abundance and bacterial activity. The study also showed that nTiO_2_ significantly affected natural lake water bacteria and the effects varied among communities from lakes depending on the chemical parameters of the water.

To study the microbiological impact of zero-valent iron in the remediation of groundwater exposed to a trichloroethylene-degrading anaerobic microbial community Zabetakis et al. [25] monitored the changes in total bacterial and archaeal population numbers using qPCR.

Londono et al. [15] spiked TiO_2_ at 700 µg/L, ZnO at 70 µg/L either separately or combined to freshly collected river water samples to investigate effects of engineered nanoparticles (ENPs) at environmentally relevant concentrations to aquatic microbial communities. DNA extraction was employed followed by next-generation sequencing to understand the change of microbial distribution responding to the presence of the ENPs in river water samples. Species richness (S) was determined by a genera or species taxa count, while for each treatment the Shannon index (H) for species diversity and species evenness was calculated. Compared to controls where no ENPs were added, the addition of TiO_2_ NPs alone at the tested concentration had no statistically significant effect on both the bacterial and eukaryotic communities. The presence of added ZnO or ZnO + TiO_2_ led to significant shift of the microbial community structure and genus distribution. This shift was more obvious for the bacteria than the eukaryotes. Based on results from single particle–inductively coupled plasma–mass spectrometry (SP-ICP-MS), all ENPs aggregated rapidly in water and resulted in much larger particles sizes than the original counterparts. The “dissolved” (including particles smaller than the size detection limits and dissolved ions) concentrations of Ti and Zn also increased in the treatment groups vs. the controls [15].

Most of the conducted studies demonstrated that natural aquatic bacterial communities were more sensitive towards individual MONPs than single strains, due to differences in resistance, highlighting the importance of their investigation [11,15]. Comparing the effect of nTiO_2_ on complex bacterial communities with the effect on single species in other studies, Farkas et al. [11] assumed that in complex bacterial communities the sum of effects on different strains will determine the overall effect on bacterial abundance and productivity. However, in the case of bacterial communities the factors that are toxic to a subset of the community might affect negatively the functioning of the overall community, altering the interaction among the strains [2].

To study microbial diversity in the aquatic environment several biochemical or molecular techniques emerged, as seen in the articles cited above. However, the application of the Biolog EcoPlate™ for studying the MONPs-mediated influences on microbial diversity in aquatic systems was demonstrated only in a few studies. In spite of this, Biolog EcoPlate™ is a low-cost, convenient and rapid technique for investigating the physiological diversity in the environment and provides information about community-level physiological profiles (CLPP).

Christian and Lind [26] explored several aspects of Biolog EcoPlate™ use for freshwater bacterial assemblages including inoculum density, incubation temperature, non-bacterial colour development, and substrate selectivity and proposed a technique to allow EcoPlate™ use in anaerobic freshwater bacterial studies.

Xie et al. [27] recently explored bacterial diversity, community composition and metabolic function in Lake Tianmuhu, China, and in its upstream dammed river, using Illumina MiSeq sequencing and the Biolog EcoPlate™ method to identify the effects of anthropogenic disturbances, such as pollution discharge and damming on biodiversity.

Even though the above-mentioned studies did apply the Biolog EcoPlate™ method for soil bacterial community analysis, unfortunately, they did not report on some important parameters, such as the reagents, sample preparation, incubation time, applied dilutions and precise formulas for calculations. In addition, these studies usually only evaluated a few Biolog-derived endpoints (usually just AWCD and AUC, sometimes SR) in terms of biodiversity and microbial community structure. The majority of the conducted microbial community studies tested ENPs typically higher than 100 mg/L concentrations, and only some studies reported above 1 mg/L [15].

The objective of our work was the evaluation of the applicability of the Biolog EcoPlate™ technique, a promising tool for community-level physiological profiling, in the investigation of the concentration- and time-dependent effect of MONPs on the biodiversity and functionality of freshwater microbial communities.

The research was conducted in lab-scale aquatic microcosms including only water from Lake Balaton as a model for aquatic surface environments.

We hypothesised that: (i) the metallic NPs (nTiO_2_ and nZnO) added to Balaton water would alter the activity and community composition of microbes, (ii) the concentration of NPs as well as the exposure time would influence their effect, (iii) the applicability of Biolog EcoPlate™ for characterising the effect of metal oxide nanoparticles on freshwater microbial communities would be demonstrated based on substrate use patterns and that (iv) the applied Biolog-derived endpoints—characterising both the microbial activity and diversity—would provide reliable and a wide range of information about the microbial communities affected by NPs exposure.

## 2. Materials and Methods

### 2.1. Freshwater Samples from Lake Balaton

Water samples from Lake Balaton, Révfülöp, Hungary (46°49′36.5″ N 17°37′50.5″ E) were collected in sterile bottles and kept at 4 °C for 24 h until the start of the experiments. For the investigation of the effect of nZnO and nTiO_2_ the water samples were taken in July and October 2020, respectively. A 3000 mL freshwater sample was collected in sterile glass bottles and transported to the lab. The main physico-chemical properties of the freshwater samples from Lake Balaton collected in July and October are shown in Table 1.

### 2.2. Characteristics of the Tested Nanomaterials

The examined nanoparticles were purchased from the manufacturers: nano zinc oxide (nZnO) from Sigma-Aldrich Inc. (Budapest, Hungary) (CAS number: 1314-13-2) and AERODISP^®^ VP Disp. W2370X nano titanium dioxide (nTiO_2_) were purchased from Evonik Resource Efficiency GmbH (Essen, Germany) (CAS number: 13463-67-7). The particle size of nZnO was less than 100 nm with an average of ≤40 nm. The mean aggregate size of nTiO_2_ was ≤100 nm and the mean particle diameter by mass was 16 nm. Details and properties on the NPs were provided by the manufacturers. The NPs were sonicated for 15 min before usage.

### 2.3. Experimental Setup

Two separate series of experiments—with identical experimental setups—were run in triplicate to assess the effect of nZnO and nTiO_2_ in 100 mL microcosms. Samples of 45 mL Balaton water were transferred into sterile, 100 mL volume Schott-bottles. Then each bottle was completed to 50 mL with the studied NP suspension at different concentrations aiming to reach 0.8, 4, 20 and 100 mg/L final NP nominal concentration in each microcosm. The original NP suspensions were diluted with distilled water. Every treatment had 3 replicates. Distilled water (DW) was the control. The proportion of DW in the control microcosms was the same as in the NPs-containing microcosms.

During the experiment samples were shaken at 120 rpm and kept in the dark at room temperature (22 ± 2 °C). For monitoring the effect of NPs, the microcosms were sampled on the 3rd, 7th, and 14th days.

### 2.4. Monitoring

#### 2.4.1. Physico-Chemical Methods

The pH and electric conductivity of the freshwater samples from Lake Balaton and the assembled microcosms were determined with a WTW (Wissenschaftlich Technische Werkstätten GmbH, Weilheim in Oberbayern, Germany) instrument using a pH 330 Meter including a precision Sentix 81 pH Electrode on the initial day and the 3rd, 7th, and 14th days in three parallels. Electric conductivity (EC) was determined with a Consort C535 instrument (Turnhout, Belgium).

#### 2.4.2. Microbiological Methods

The enzymatic activity of viable cells [28,29] was measured by a tetrazolium reduction viability assay with ELISA Microplate Reader (Vienna, Austria) for assessing metabolic activity. Three 200 µL samples were introduced into the wells of a sterile microplate and the OD was measured at 630 nm.

Then 30 µL of 1 mg/mL 3-(4,5-dimethylthiazol 2-yl)-2,5-diphenyltetrazolium bromide (MTT) was transferred into every well, and the plate was incubated for 15 min in the dark at room temperature.

The absorbance was measured at 490 nm with DIALAB EL800 Microplate Reader. The inhibition percentage (I%) compared to control was calculated based on the absorbance values with the following Equation:

(1) $$I\%=\frac{C-S}{C}\cdot 100,$$

where C is the absorbance of the control and S is the absorbance of the NP containing sample [30].

#### 2.4.3. Biolog EcoPlate™

The Biolog EcoPlate™ test method [31] was applied to monitor the effect of zinc oxide and titanium dioxide nanoparticles on the microbial communities, and how they influence their substrate use (community-level physiological profiling, CLPP).

The EcoPlate™ includes three replicate wells containing 31 organic carbon substrates and a control well with redox-sensitive tetrazolium dye, but no substrate, for community-level physiological profiling (CLPP) of metabolically active heterotrophic bacterial assemblages able to grow in plate conditions [32]. Biolog-derived data for evaluation from CLPP include: the average well colour development (AWCD) and the Shannon diversity index (H) [32,33,34]. AWCD shows the general potential metabolic activity of the microbial community, thus it indicates the total bioactivity of the Biolog EcoPlate™ [33]. The Shannon diversity index (H) informs on the physiological diversity of bacterial communities [33]. Higher values of H indicate metabolically active microbial communities able to degrade more substrates [34]. Additional Biolog EcoPlate™-derived parameters include substrate richness (SR) and Shannon evenness (E) [35].

Although the Biolog EcoPlate™ methodology does not inform about the genetic composition of the microbial community, it provides valuable insight into bacterial substrate use patterns and metabolic functional potential in environmental matrices affected by stressing factors, such as contamination. In addition, it could be applied as a monitoring method, since it is rapid, low cost, reproducible and simple.

Therefore, this study aims to provide evidence to the applicability of the Biolog EcoPlate™ technique for the characterisation of the microbial activity and biodiversity of nTiO_2_- and nZnO-impacted freshwaters. We compared several endpoints, primarily those associated with the effects on biodiversity and microbial community structure. The conventional endpoints, such the AWCD, the substrate richness (SR), the area under the curve (AUC) and substrate average well colour development (SAWCD) were analysed, but microbial diversity and its changes triggered by stress factors were in focus. The endpoints studied and compared in terms of microbial diversity included: Shannon diversity index, Shannon evenness index, Simpson index, McIntosh index and Gini coefficient.

Measurements were carried out by pipetting 125 µL samples into each well of the EcoPlate. Plates were incubated in the dark at 25 °C, and the absorbance of the wells was measured every 24 h for 120 h at 490 nm wavelength by DIALAB EL800 Microplate Reader. Although the Biolog EcoPlate™ method usually measures optical density (OD) at 590 nm, because the highest absorbance of the tetrazolium dye could be measured at this wavelength [31,34,36], our microplate reader was equipped with 405, 450, 490 and 630 nm filters, and the optimal OD values were provided at 490 nm [37,38].

Because the 96 wells of the Biolog EcoPlate™ contain 31 carbon sources and control wells (blank) in 3 replicates, all measurements were done 3 times, 1–1 series from the 3 replicates of the experimental setup.

The measured absorbance values were corrected before evaluation. Firstly, the OD values of the control wells (which contain water to read the clear absorption value) were subtracted from the substrate containing wells’ OD values. Next, the initial OD value of each well, measured immediately after pipetting the suspension into the wells, was subtracted from each OD value corrected in the previous step. The aim was to eliminate the background effect (the effect of nanoparticles on the optical density values). OD values measured at 120 h were used for data evaluation because these indicated the optimal range of OD readings [35,37,39]. Different endpoints were calculated from the corrected data including average well colour development (AWCD), substrate average well colour development (SAWCD), substrate richness (SR), Shannon index (H), Shannon evenness (E), Simpson index (D), McIntosh index (U), and Gini coefficient (G).

Average well colour development (AWCD) was computed by the following equation for all carbon sources:

(2) $$\mathrm{AWCD}=\frac{\sum_{i=1}^{N}{\mathrm{OD}}_{i}}{N}$$

where OD_i_ is the corrected OD value of each substrate-containing well, while N is the number of substrates, which in this case is N=31 [35,38]. For further investigation of the AWCD, all carbon substrates were classified into six categories based on their characteristics according to Sala et al. [40]: polymers (alpha-cyclodextrin, glycogen, Tween 40, Tween 80), carbohydrates (D-cellobiose, i-erythritol, D-galactonic acid-gamma-lactone, N-acetyl-D-glucosamine, glucose-1-phosphate, beta-methyl-D-glucoside, D,L-alpha-glycerol phosphate, alpha-D-lactose, D-mannitol, D-xylose), carboxylic acids (gamma-hydroxybutyric acid, alpha-ketobutyric acid, D-galacturonic acid, D-glucosaminic acid, itaconic acid, D-malic acid, pyruvic acid methyl ester), amino acids (L-arginine, L-asparagine, glycyl-L-glutamic acid, L-phenylalanine, L-serine, L-threonine), amines (phenylethylamine, putrescine), and phenolic compounds (2-hydroxybenzoic acid, 4-hydroxybenzoic acid). For every substrate category the substrate average well colour development (SAWCD) value was calculated by the following equation:

(3) $$\mathrm{SAWCD}=\frac{\sum_{i=1}^{N}{\mathrm{OD}}_{i}}{N},$$

where OD_i_ is the corrected OD value of the substrates within the substrate category and N is the number of substrates in the category [33].

Substrate richness (SR) is the number of oxidised substrates. It was computed as the total of the cells’ OD_i_ value which was at least 0.5 after 120 h incubation [41].

The area under the curve (AUC) was computed by the following equation [42]:

(4) $$\mathrm{AUC}=\sum_{i=1}^{N}\sum_{j=1}^{n}\frac{{\mathrm{OD}}_{i_{n}}+{\mathrm{OD}}_{i}{}_{n+1}}{2\;\cdot \;\left(t_{i_{n+1}}-t_{i_{n}}\right)},$$

where N is the number of substrates, n is the number of measurements, OD_i_ is the corrected OD value of each individual well, at two consecutive measurements at two different measurement times for t_n_ and t_n + 1_.

Shannon index (H) was calculated according to the following equation:

(5) $$H=-\overset{N}{\underset{i=1}{\sum }}P_{i}\cdot \ln P_{i}$$

where N is the number of substrates, P_i_ is the relative colour development of the well over the total colour development of each well of a plate [34,43,44].

(6) $$P_{i}=\frac{{\mathrm{OD}}_{i}}{\sum_{i=1}^{N}{\mathrm{OD}}_{i}}$$

Shannon evenness (E) was derived from the Shannon index by its partition based on the substrate richness according to Jałowieczki et al. [42]:

(7) $$E=\frac{H}{\ln\left(\mathrm{SR}\right)}\;,$$

where H is the computed Shannon index, while SR is the calculated substrate richness.

Simpson index (D) was computed by the following equation:

(8) $$D=-\ln\sum_{i=1}^{N}{\left(P_{i}\right)}^{2},$$

where Pi and N are the same as in the Shannon index calculation [45].

McIntosh index (U) was calculated according to Manjunath et al. [46]:

(9) $$U=\sqrt{\sum_{i=1}^{N}{\left({\mathrm{OD}}_{i}\right)}^{2}},$$

where N is the number of substrates, OD_i_, is the corrected OD value of each substrate containing well. 

Gini coefficient (G) was calculated by the following equation:

(10) $$G=\frac{\sum_{i=1}^{N}\sum_{j=1}^{N}\left|{\mathrm{OD}}_{i}-{\mathrm{OD}}_{j}\right|}{2N^{2}\;\cdot \;\left(\mathrm{AWCD}\right)}\;$$

where N is the number of substrates, OD is the corrected OD value of each well, AWCD is the computed AWCD value as described previously [32].

#### 2.4.4. Statistical Analysis of Data

Data were submitted to analysis of variance (ANOVA) using StatSoft^®^ Statistica 13.1 (TIBCO Software, Inc., Palo Alto, CA, USA) to calculate if the effect of a treatment was significant. For this purpose, two types of statistical analyses were done, depending on the examination.

To investigate the significant differences with respect to enzymatic activity determined by MTT, one-way ANOVA was used. To verify the criteria for the homogeneity of variances Cochran’s C test was applied.

Repeated measures ANOVA (RMANOVA) was used to investigate the significant effects of the nZnO and nTiO_2_ treatments with Biolog EcoPlate™ tests. The Mauchley sphericity test was applied to confirm the criteria.

Both types of statistical analyses were performed at the *p* < 0.05 significance level. Fisher’s Least Significant Difference (LSD) was used for comparison of the effects of the various treatments. The significant effects are marked with letters on all figures in alphabetical order, where “a” is the smallest average value. Columns signed with the same letter indicate that there was no significant difference between them.

All measured microcosm data were subject to Pearson correlation analysis for detecting any coherence between the concentration of the applied NPs and diversity indices.

## 3. Results

### 3.1. Physico-Chemical Characteristics of Lake Balaton

The pH and electrical conductivity of the water samples did not change significantly compared to control after 14 days. In the case of the nTiO_2_ experimental series, the pH was 8.69 ± 0.03 and the EC was 632.0 ± 9.5 µS, while in the nZnO experimental series the pH was 8.97 ± 0.12 and the EC was 693.8 ± 32.9 µS after 14 days.

### 3.2. Effect of TiO_2_ and ZnO Nanoparticles on Enzymatic Activity

The results of the enzymatic activity test based on the tetrazolium reduction viability assay presented low sensitivity (data not shown).

TiO_2_ NPs triggered no significant inhibition (<5%), while ZnO NPs exhibited medium but concentration-dependent inhibition of the enzymatic activity. ZnO at 100 mg/L concentration resulted in the highest inhibition (60 ± 21%), followed by the lower nZnO doses such as 20 mg/L, 4 mg/L and 0.8 mg/L nZnO, which caused 48 ± 20%, 23 ± 5% and 12 ± 1% inhibition compared to control, respectively.

### 3.3. Effect of ZnO Nanoparticles on the Community-Level Physiological Profiles (CLPP)

**Figure 1:**
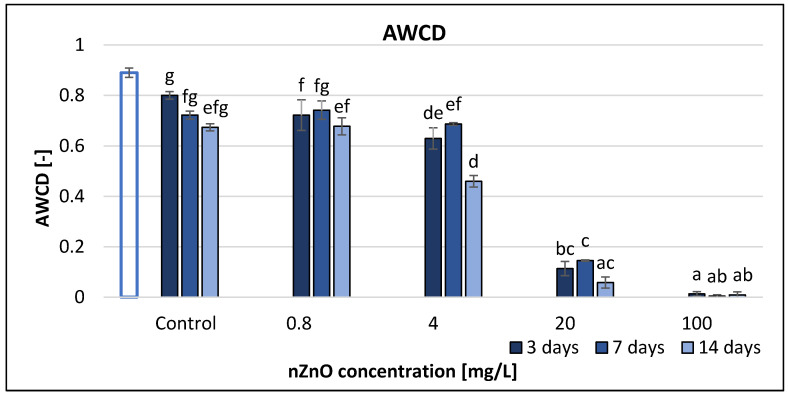
The effect of ZnO nanoparticles on freshwater average well colour development value. Letters on the columns indicate significant differences (level of significance: *p* < 0.05).

Average well colour development (AWCD) significantly decreased from the start to the end of the experiment. The different nZnO concentrations had a significant effect on the AWCD, but the interaction of time and nZnO treatment in different concentrations did not (Table 2). According to Figure 1, 100 mg/L nZnO had the highest effect (98–99% inhibition), while 0.8 mg/L nZnO the smallest (11–58%). However, the lowest nZnO concentration (0.8 mg/L) resulted in a significant decrease compared to the control in the short term (after 3 days). nZnO had significant impact at 4, 20 and 100 mg/L nZnO concentrations after 14 days. Correlation analysis (Table S1) indicated strong linear negative association between ZnO NP concentration and AWCD at each sampling time (*r* = −0.99).

**Figure 2:**
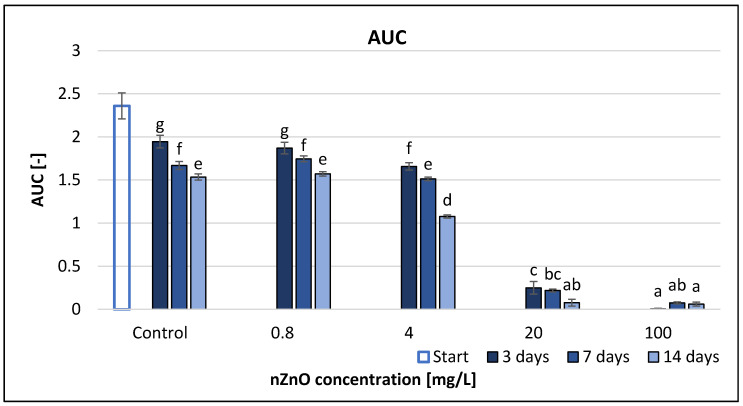
The effect of ZnO nanoparticles on freshwater area under the curve value. Letters on the columns indicate significant differences (level of significance: *p* < 0.05).

The area under the curve (AUC) had a similar trend to AWCD (Figure 2), but in this case the interaction of treatment and time also had a significant effect, similarly to the separate effect of time and treatment (Table 2). There was a decreasing trend in the detected AUC values at the same concentration on different sampling days, except for the 100 mg/L nZnO concentration, which showed a slight growth. However, the increasing nZnO amount significantly reduced the AUC values (Figure 2). The nZnO concentrations of 4 mg/L and above exhibited significant difference compared to control at each measurement time. The results of the correlation analysis (Table S1) showed that the AUC values negatively correlated (*r* = −0.98–1.00) with the nZnO concentration after 3, 7 and 14 days of exposure (*p* < 0.05).

**Figure 3:**
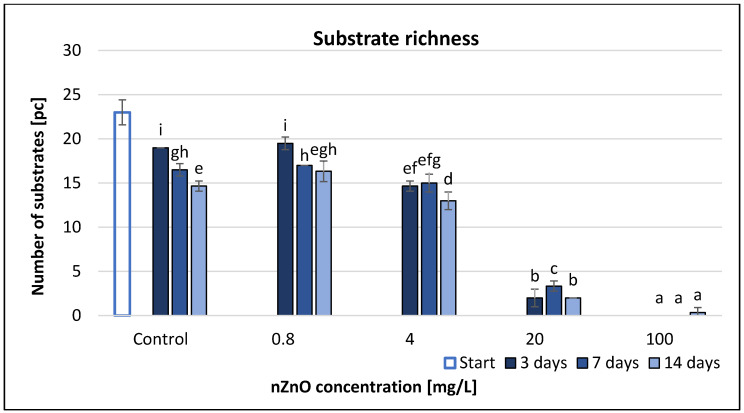
The effect of ZnO nanoparticles on freshwater substrate richness value. Letters on the columns indicate significant differences (level of significance: *p* < 0.05).

The effect on substrate richness (SR) was similar to that on the AUC values (Figure 3). The high nZnO concentrations significantly decreased the usage of many substrates. A concentration of 100 mg/L nZnO resulted in no detectable substrate use on the 3rd and 7th day of the experiment, while on the 14th day a colour change occurred in one substrate (pyruvic acid methyl ester), which was consistent with the results of AUC. Time, treatment and their interaction had significant effect on the SR values based on RMANOVA analysis (Table 2). The correlation analysis (Table S1) showed strong and negative correlation between the nZnO concentration and the SR values (*r* = −0.97–0.99) after 3, 7 and 14 days of exposure (*p* < 0.05).

According to our results, Shannon index (H) had similar structure to the AWCD, AUC and SR pattern, although it seemed to be less sensitive at low concentrations, and it had a peak value at 20 mg/L nZnO concentration.

**Figure 4:**
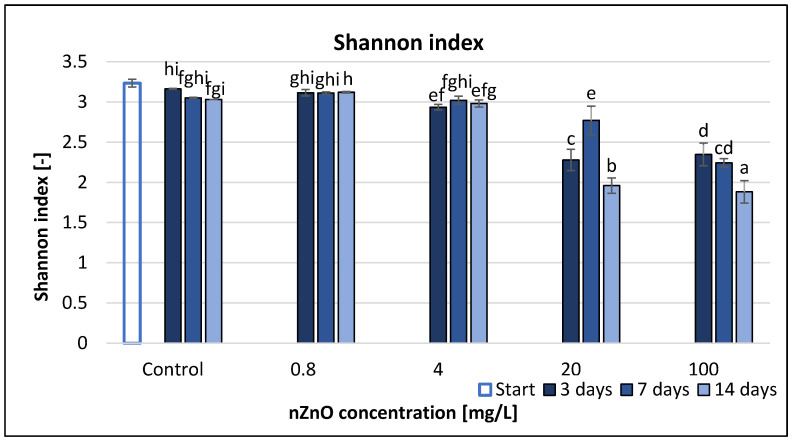
The effect of ZnO nanoparticles on freshwater Shannon index value. Letters on the columns indicate significant differences (level of significance: *p* < 0.05).

On the other hand, the difference between the H values at 20 and 100 mg/L nZnO was not as high as in case of the previous endpoints (AWCD, AUC and SR) at these concentrations. In addition, the H values at 20 and 100 mg/L nZnO were significantly lower than in case of lower nZnO concentrations (Figure 4). RMANOVA demonstrated the significant effect of time, treatment and their interaction (Table 2).

The correlation analysis (Table S1) displayed a strong significant correlation between the nZnO concentration and the H values (*r* = −0.99) only after 3 and 14 days exposure (*p* < 0.05).

**Figure 5:**
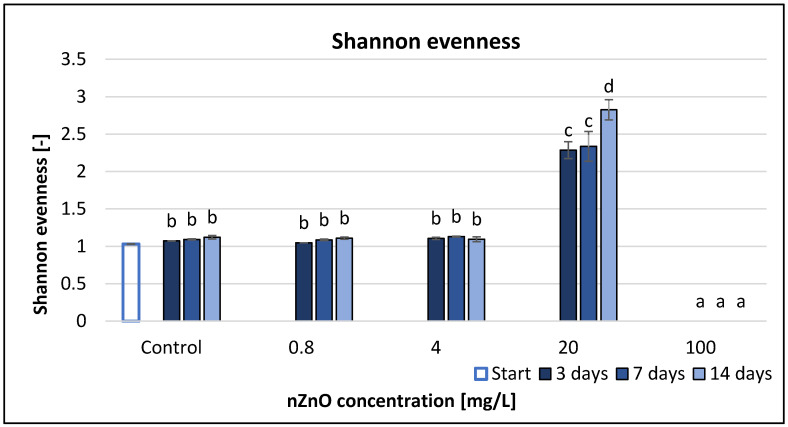
The effect of ZnO nanoparticles on freshwater Shannon evenness value. Letters on the columns indicate significant differences (level of significance: *p* < 0.05).

Shannon evenness (E), derived from the Shannon index (H), did not show any significant effects compared to the control at the smallest applied nZnO concentrations (0.8 and 4 mg/L). However, the E value at 20 mg/L nZnO concentration indicated significant effect (Figure 5). The relevant Equation (7) and the involved SR values for the calculation of E did not result any E values at 100 mg/L nZnO. According to the RMANOVA statistical analysis, only the treatment with ZnO nanoparticles had significant effect (Table 2). The Pearson correlation analysis (Table S1) displayed significant strong correlation between the nZnO concentration and the E values but only after 7 and 14 days of exposure (*r* = −0.723 and −0.614 at *p* < 0.05, respectively).

**Figure 6:**
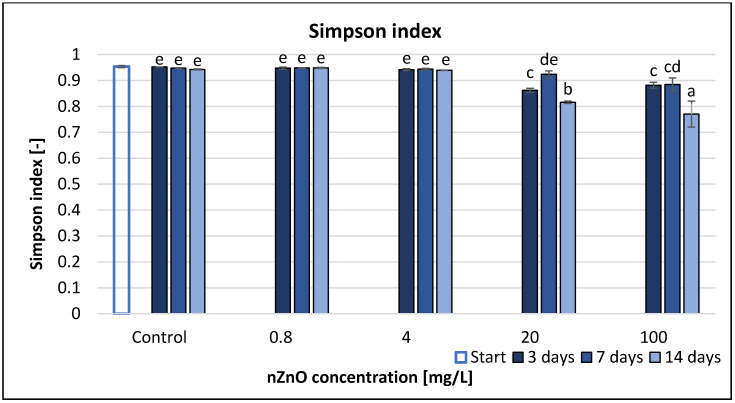
The effect of ZnO nanoparticles on freshwater Simpson index value. Letters on the columns indicate significant differences (level of significance: *p* < 0.05).

Based on the Simpson index (D), similarly to the Shannon evenness, ZnO nanoparticles at 0.8 and 4 mg/L concentration had no significant effect compared to the control (Figure 6). The detectable significant effect occurred only at 20 and 100 mg/L concentrations; the highest decrease (18%) occurred at 100 mg/L nZnO concentration after 14 days. Although the highest tested concentration caused significant decrease compared to control at each sampling time, RMANOVA analysis illustrated that the time, treatment and their interaction had a significant effect on the Simpson index values (Table 2). Correlation analysis with coefficients *r* = −0.641 (3rd day), −0.887 (7th day) and −0.849 (14th day) illustrated significant linear negative relationship between the concentration and Simpson index (Table S1).

**Figure 7:**
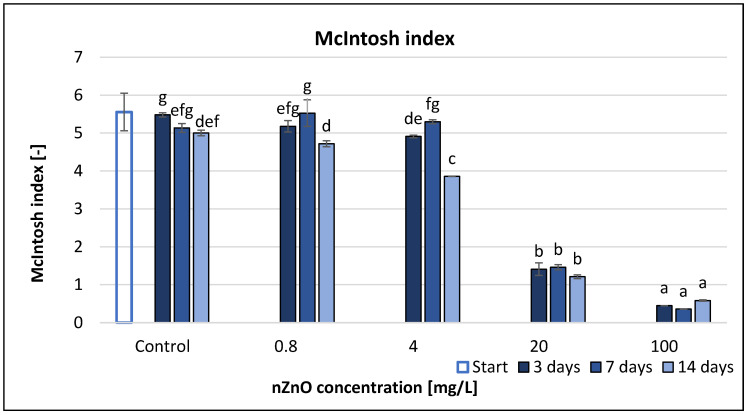
The effect of ZnO nanoparticles on freshwater McIntosh index value. Letters on the columns indicate significant differences (level of significance: *p* < 0.05).

McIntosh index (U) was the most sensitive diversity index for indicating the effect of ZnO nanoparticles on microbial community according to Figure 7. Generally, the U values decreased with the increase of the applied nZnO concentrations and the elapsed time of the experiment. 20 mg/L nZnO concentration decreased the McIntosh index by 74–76% compared to the non-treated control, while 100 mg/L nZnO resulted in 90–92% reduction. The RMANOVA clearly illustrated the very strong significant effect (*p* value 0.000) of treatment and time, and even their interaction (Table 2.). Correlation analysis with coefficients r = −0.835 (3rd day), −0.838 (7th day) and −0.806 (14th day) illustrated strong linear negative relationship between the concentration and Simpson index, which was significant at *p* < 0.05 (Table S1).

**Figure 8:**
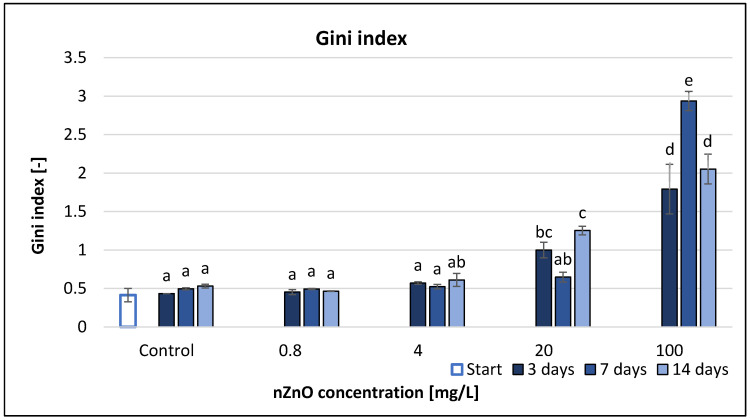
The effect of ZnO nanoparticles on freshwater Gini index value. Letters on the columns indicate significant differences (level of significance: *p* < 0.05).

The Gini index (G) increased with the applied nZnO concentration (Figure 8). Low Gini index values indicated high levels of functional diversity [47]. Gini index illustrated significant effects only at higher nZnO concentrations (20; 100 mg/L). Highest increase (493%) compared to control occurred at 100 mg/L concentration after 7 days. Statistical analysis showed that not just the treatment, but also the treatment-time interaction had a significant effect (Table 2). Correlation analysis (*r* = 0.824–0.903) revealed strong positive relationship between the concentration and Gini index, which was significant at *p* < 0.05 (Table S1).

**Figure 9:**
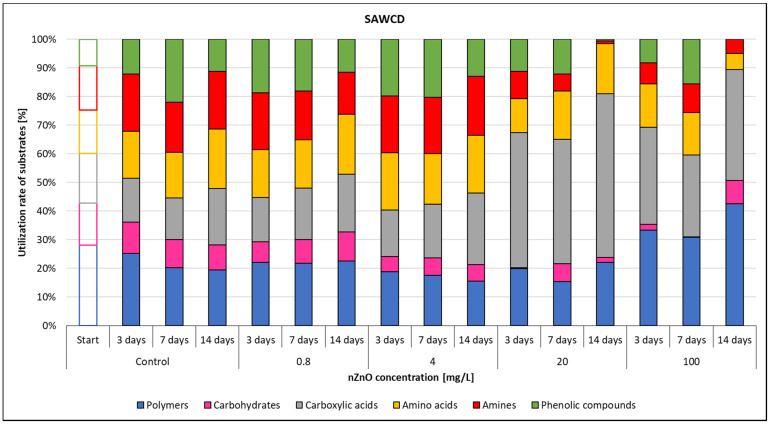
The effect of ZnO nanoparticles on freshwater substrate average well colour development value.

The microbial metabolic pathways were also influenced by the ZnO nanoparticles, as confirmed by the substrate average well colour development values. The use ratio of each substrate category was illustrated in Figure 9, considering that total use of the six different substrate groups was 100%. The SAWCD ratio by substrate groups was highly influenced by nZnO concentration. The metabolism of polymers decreased with the elapsed days and the applied nZnO concentration, although, at 100 mg/L nZnO concentration the ratio of the total substrate use increased.

Use of amines had a similar pattern. At 20 mg/L nZnO dose the amine metabolism decreased, similarly to the phenolic compounds. On the other hand, the use ratio of carboxylic acids increased with the applied nZnO concentration, suggesting that use of these substrates was not influenced by ZnO nanoparticles, or it was intensified by the changed environmental parameters.

According to the results, the use of each substrate group was influenced by the presence of nZnO especially at 20 and 100 mg/L concentrations.

Although carbohydrates were the most represented substrate class in Biolog EcoPlate™, they were the least used (0–14.5%)—while polymers (15–43%) and carboxylic acids (15–56%) had the highest contribution to CLPP.

### 3.4. Effect of TiO_2_ Nanoparticles on the Community-Level Physiological Profiles (CLPP)

**Figure 10:**
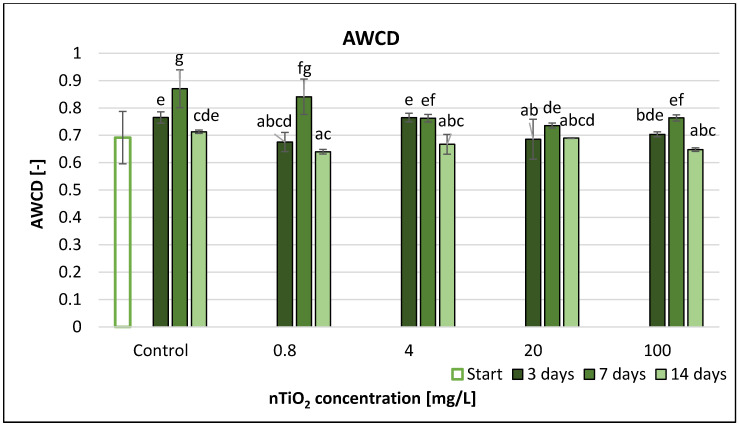
The effect of TiO_2_ nanoparticles on the freshwater average well colour development value. Letters on the columns indicate significant differences (level of significance: *p* < 0.05).

The average well colour development (AWCD) values usually showed a slight decline from the start to the end of the experiment (Figure 10) such as AUC values (Figure S1). The highest decrease (15%) was measured at 20 mg/L nTiO_2_ concentration after 7 days. A concentration of 100 mg/L nTiO_2_ resulted in 9% decrease compared to control after 14 days. However, the extent of the decrease was low. The RMANOVA analysis demonstrated that nTiO_2_ treatment and time as well as their interactions had significant effect on the AWCD (Table 3). The most significant changes were after 1 week, with significant differences between the AWCD values in the control and the nTiO_2_ microcosms mainly at higher concentrations (4–100 mg/L). However, after 2 weeks, nTiO_2_ displayed no significant effect. Correlation analysis did not show a clear correspondence between concentration and AWCD (Table S2).

**Figure 11:**
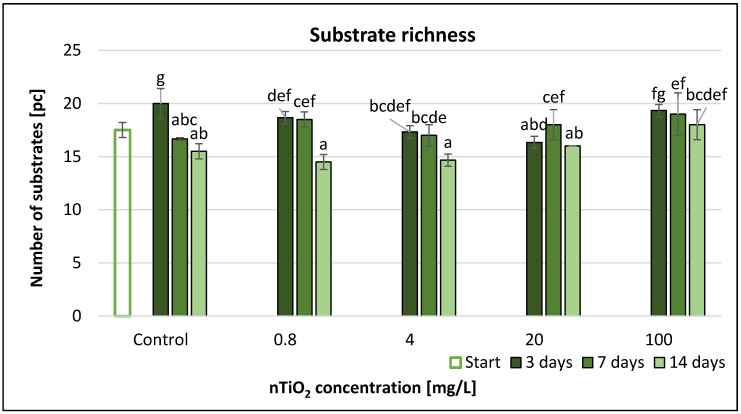
The effect of TiO_2_ nanoparticles on freshwater substrate richness value. Letters on the columns indicate significant differences (level of significance: *p* < 0.05).

The evolution of the substrate number (SR) values function of concentration had different trends at each sampling time (Figure 11). At up to 20 mg/L nTiO_2_ concentration it decreased continuously after 3 days; however, at 100 mg/L concentration, there was no significant change compared to control. A small increase was encountered proportionally with the concentration after both 7 and 14 days of exposure. The largest difference (~19% inhibition) versus the control occurred at 20 mg/L concentration level, after 3 days. The RMANOVA (Table 3) demonstrated that the treatment did not influence the evolution of substrate number; but the effect of time and the interactive effects of time × treatment were significant on SR. Correlation analysis showed strong positive correlation between concentration and SR (*r* = 0.850) after 2 weeks of exposure (Table S2).

**Figure 12:**
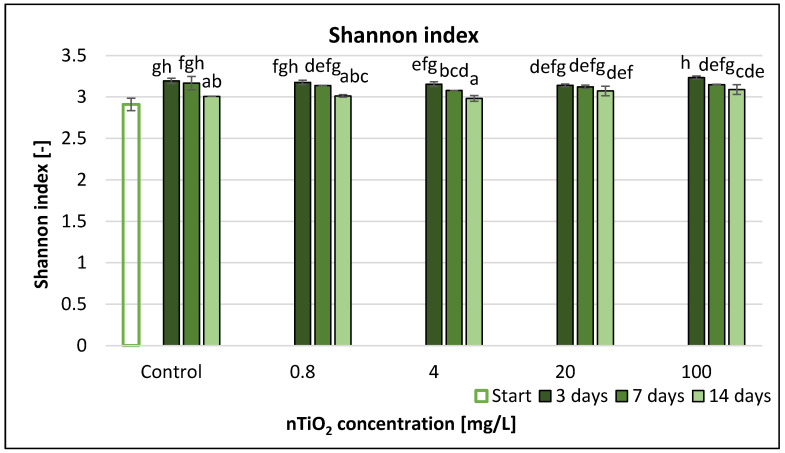
The effect of TiO_2_ nanoparticles on freshwater Shannon index value. Letters on the columns indicate significant differences (level of significance: *p* < 0.05).

The Shannon index (H), applied widely for characterising microbial diversity, showed only very small differences both in time and function of nTiO_2_ doses (Figure 12). Significant changes compared to control occurred at 4 mg/L nTiO_2_ after 7 days and at 20 and 100 mg/L nTiO_2_ after 14 days.

The highest alteration (less than 5%) compared to control occurred at 4 mg/L nTiO_2_ after 7 days. Based on the RMANOVA results, TiO_2_ nanoparticles (treatment) and time had significant effects on the Shannon index (Table 3). Pearson correlation coefficients did not indicate significant correlation between variables in this case (Table S2), but Shannon evenness (Figure S2) had significant correlation after 14 days exposure (Table S2).

**Figure 13:**
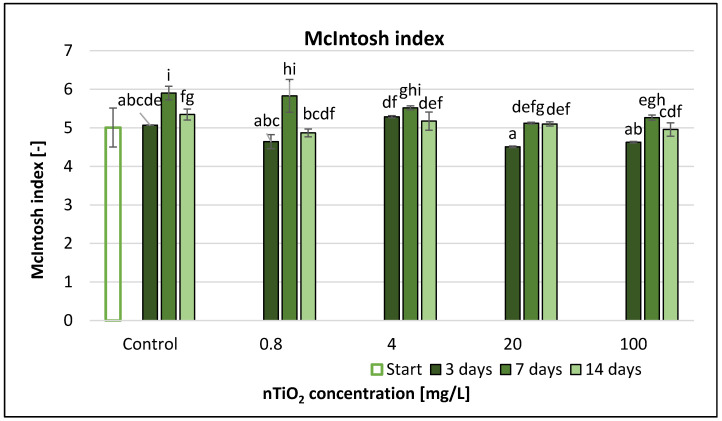
The effect of TiO_2_ nanoparticles on freshwater McIntosh index value. Letters on the columns indicate significant differences (level of significance: *p* < 0.05).

The McIntosh index (Figure 13) showed higher differences both in time and as a function of nanomaterial concentration compared to the Simpson index (Figure S3). However, these changes did not exhibit a clear tendency function of nTiO_2_ concentration.

Significant differences compared to the control occurred only at 20 and 100 mg/L nTiO_2_ after 7 days and the McIntosh index values were the highest at this time point. During the 7th day, the McIntosh index values showed a slight decreasing trend with the increase of nTiO2 concentration. The largest decrease compared to control occurred at 20 mg/L and 100 mg/L, representing 13% and 11%, respectively.

Based on the results of correlation analysis, there was only a slight non-significant association between the two variables (TiO_2_ concentration and McIntosh index) (Table S2), such as in case of Gini index (Figure S4).

The RMANOVA analysis of the Biolog EcoPlate™ data based on the McIntosh index showed a clear significant effect of treatment and time, as well as of their interaction (Table 3).

**Figure 14:**
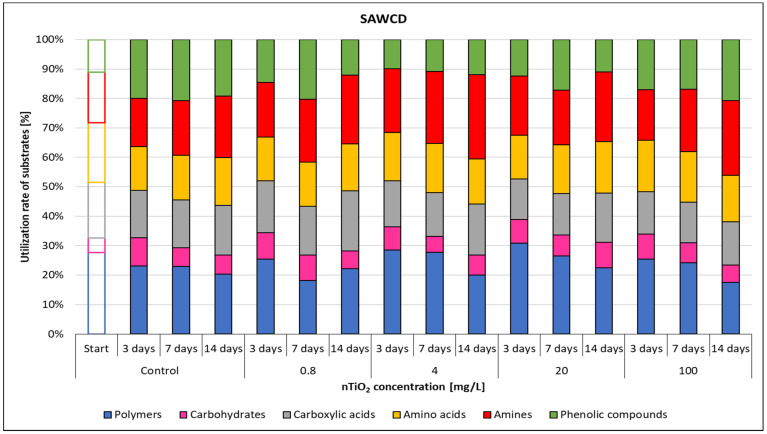
The effect of TiO_2_ nanoparticles on freshwater substrate average well colour development value.

The SAWCD ratio per guild in the nTiO_2_-contaminated microcosms did not change significantly during the experiment (Figure 14).

According to the percent distribution of SAWCD for each nTiO_2_ concentration (Figure 14), the use rate of the substrate groups did not change significantly with incremental nTiO_2_ concentrations.

The microbes in the different microcosms used all six substrate groups, but there were differences in the use of the various groups regardless of nTiO_2_ concentration and elapsed time.

The use rate of carbohydrates (5–10%) was the lowest, while those of polymers (18–31%) and amines (16–29%) were the highest.

## 4. Discussion

Currently, the amount of available research data concerning the effect of metal oxide nanoparticles on freshwater bacterial communities is very limited. The results of the present study reveal the necessity of assessing the impact of nano-metal oxides not only on single organisms from higher trophic levels, but also on microbial communities in aquatic systems. The results demonstrated that the effects of the two tested MONPs (nTiO_2_ and nZnO) on the freshwater microbial systems were different. Although both the tested concentrations and the exposure times were identical in the two freshwater ecosystems, the Biolog EcoPlate™ system identified significant differences between their effects.

### 4.1. Assessment of the Impact of TiO_2_ Nanoparticles on Freshwater Microbial Diversity

TiO_2_ NPs had a low, but significant effect on microbial communities. This result correlates with Miao et al.’s [48] about the effect of TiO_2_ and cerium dioxide (CeO_2_) NPs on the ecosystem of aquatic sediments. They found that oxygen consumption decreased in the case of both NP types, but nTiO_2_ had a less acute toxic effect compared to nCeO_2_. Although the treatment increased bacterial diversity, the metabolic activity of benthic microbial communities was negatively affected by the NPs [48]. Our results were in accordance with Londono et al. [15] and Chavan and Nadanathangam [49], who found that the addition of TiO_2_ NPs had a lower effect on the bacterial communities than the nZnO under the same experimental parameters. According to several studies, TiO_2_ nanoparticles in surface waters and aquatic test systems may form agglomerates, so the resulting TiO_2_-induced effect is due to the presence of agglomerates, in addition to individual and well-dispersed primary NPs [13,50]. Thus, microorganisms in surface waters may be exposed to NPs of different form and particle size. The typical nTiO_2_ toxicity mechanism is the formation of reactive oxygen species (ROS) through photocatalytic reaction, followed by a subsequent damage of the lipid membrane [13]. However, our experiments were performed in the dark, so this pathway could not play a significant role, which may explain the slighter effect on the microbial community. Since TiO_2_ NPs are not expected to dissolve under the studied conditions, other mechanisms in the bacterial community may be responsible for the low-level but significant effect of nTiO_2_, such as enhanced membrane permeability and subsequent penetration of nanoparticles, in addition to the adsorption of NPs to the cell membrane which may induce intracellular ROS [13]. Additionally, the binding affinity of NPs to organic molecules, the penetration of primary particles (<10 nm) through damaged membranes and matrix effects may induce varying degrees of toxicity [13,50,51].

### 4.2. Assessment of the Impact of ZnO Nanoparticles on Freshwater Microbial Diversity 

In line with studies about the effects of nanomaterials in aquatic and terrestrial systems performed with different methods we found a concentration- and time-dependent effect of nZnO on bacterial community [15,49,52].

ZnO nanoparticles significantly and stably inhibited leaf decomposition in stream water at 100 mg/L throughout a 50-day exposure, as described by Du et al. [53]. Inhibition occurred only on the 43rd day at 10 mg/L dose of nZnO, but fungal diversity decreased. According to Du et al. [54], ZnO NPs increased the fungal biomass in aquatic ecosystems after 5 days of exposure, but after 45 days, chronic effects caused a significant decrease even at 300 ng/L concentration. Our results were similar to Du et al.’s [54] findings, but the Biolog EcoPlate™ technique was already able to identify the negative effects of ZnO NPs after 3 days’ exposure at 0.8 mg/L. Both ZnO nanoparticles and the dissolved Zn^2+^ ions may be responsible for the significant inhibitory effect. In our study, it is unknown whether dissolved zinc or the ZnO NPs exerted the adverse effects, but this was not the aim of our research. Our results harmonised with Kusi et al.’s [55] about the negative effects of silver nanoparticles (AgNPs) on freshwater microbial activity. Kusi et al. [55] applied silver nanoparticles (AgNPs) at 125 mg/L maximum concentration in microcosms with stream water. The microbial catabolic activity decreased, resulting in a decrease of the AWCD and SR values. The change in the used carbon source pattern of Kusi et al.’s study was similar to our results regarding the substrate average well colour development (SAWCD), suggesting amino acids, carbohydrates and carboxylic acids as carbon sources for metabolic fingerprint. In our research, the most affected substrate group in terms of use, affected by nZnO was the carboxylic acids group. The substrate average well colour development (SAWCD) ratio by groups indicated significant and high nZnO-induced changes; use of all substrate groups was influenced by nZnO especially at the highest concentrations (20–100 mg/L).

The highest shift occurred in the use of carboxylic acids; the initial use (15–17%) in the control increased to 30–57% at 20 and 100 mg/L nZnO level. This was in accordance with Teng et al. [56] and Melita et al. [57], who also found that carboxylic acids were sensitive carbon sources. These substrates are products of organic matter degradation in surface waters; therefore, carboxylic acids belong to the labile organic matter sources of the aquatic environment.

The reason for the enhanced carboxylic acid use may be the consumption of easily available substrates stimulated by the degraded environmental conditions at 20 and 100 mg/L nZnO.

### 4.3. Comparison of the Effects of TiO_2_ and ZnO Nanoparticles on Freshwater Microbial Diversity

Based on the observed effects of TiO_2_ and ZnO nanoparticles, these NPs are harmful for the microbial activity. ZnO NPs had a stronger effect than TiO_2_ NPs on all the tested endpoints. In accordance with several studies, our results determined by Biolog EcoPlate™ clearly reflected that the impact of MONPs on microbial community were largely determined by the type of NP [15,49].

Most of the computed endpoints presented a significant decrease at all examined concentrations, in some cases there were even detectable significant effects at a dosage of 0.8 mg/L. The influence of ZnO NPs was correlated with exposure time and the applied concentration; thus, at 100 mg/L dosage, the substrate use activity was highly reduced after 14 days. These results are similar to the observations of Londono et al. [15], who found that nZnO had a higher influence on water bacterial community than nTiO_2_. In line with their results, nTiO_2_ did not cause significant effect at 700 µg/L, while nZnO shifted the microbial community structure and genus distribution even at 70 µg/L concentration. Similar to these results, Chavan et al. [49] reported that the metabolic diversity and activity of microbial communities in soils was impacted in the presence of zinc oxide, but not of titanium dioxide NPs.

According to the Pearson correlation results, nTiO_2_ concentration had a low correlation to the endpoints and most of them were not significant (*p* < 0.05), but the Pearson’s “*r*” values in most cases were higher with the elapsed days (Table S2), which also indicated the significance of the exposure time. In contrast to the TiO_2_ nanoparticles, almost every endpoint (except Shannon evenness) showed a high correlation with the applied nZnO concentration (|*r*| > 0.80), and most of them were significant (*p* < 0.05) (Table S1) regardless of the exposure time.

The higher damage caused by ZnO NPs to microbial activity may be due to the presence of dissolved zinc ions. Uptake of toxic dissolved zinc ions may result in decreased enzyme activity as reported by Sirelkhatim et al. [58]. According to their study, ZnO NPs caused inhibition of the respiratory enzymes [58]. Our findings may reveal this mechanism as well, as the tetrazolium reduction assay in our study displayed a concentration-dependent inhibition of enzyme activity; therefore, the inhibition mechanism may be similar to that identified by Sirelkhatim et al. [58].

### 4.4. Characterisation of the Applicability of the Biolog EcoPlate™ for the Evaluation of the Effects of Nanoparticles on Freshwater Microbial Diversity

Biolog EcoPlate™ is a low-cost, simple and easy technique to describe and compare bacterial communities and their activity. It provides information through different calculated endpoints derived from the optical density values. Because the examined nTiO_2_ concentrations proved to have significant effects only in some cases, the conclusions of our study focused mainly on the effect of nZnO. Based on our results, the AWCD, AUC and SR values significantly decreased with incremental ZnO NPs concentrations. These endpoints demonstrate the harmful effects of nZnO, but they are not completely independent from each other.

SR and AWCD interact with each other: a high AWCD value indicates use of many carbon sources, a medium AWCD value suggests that all substrates are slightly used or some carbon sources are well used and the others are not used [59]. AUC values are also calculated based on ODs, and thus the AUC has a similar pattern to AWCD and SR. RMANOVA analysis revealed significant changes in the metabolic profiles of the microbial community according to the above-mentioned indices and in the presence of ZnO nanoparticles, but significant differences were more pronounced for AUC and SR parameters.

The use rates of different substrate classes (SAWCD) have rarely been applied in Biolog EcoPlate™ studies, although they can provide important and complementary information to the frequently applied endpoints. Substrates which are promptly and preferentially used may indicate important information on the environmental conditions [57]. Interestingly, the use of carboxylic acids showed an increasing trend over time at concentrations of 20 and 100 mg/L nZnO. The use of pyruvic acid methyl ester was the most significant within the carboxylic acid group at these nanomaterial concentrations after 14 days. The use of Tween40 and Tween80 was also significant in the microcosms containing high (20 and 100 mg/L) nZnO concentrations, while the carbohydrates were used at a much lower rate than in the control. Presumably, in this impacted environment, microorganisms acquired the ability to metabolise more complex carbon sources.

Our research aimed at the comparative evaluation of the different diversity indicators such as Shannon index, Shannon evenness, Simpson index, McIntosh index and Gini coefficient. Among these, the Shannon index and Shannon uniformity are most commonly used for the estimation of functional diversity.

Shannon index provides information about the distribution of the used carbon sources. A high Shannon index implies a greater functional metabolic diversity and at the same time an even distribution of different functional types. A great evenness can only be obtained if all carbon sources are well used, but it does not work backwards. If the majority of the substrates are used, the Shannon index value will be high [59]. Based on our results, nZnO treatment decreased the values of the Shannon index. This proves the negative effects of ZnO nanoparticles on microbial activity and diversity. Shannon evenness (E) is a measure of the uniformity of metabolic activities across all substrates. Shannon evenness had lower values below 20 mg/L nZnO concentration, while at 100 mg/L there were mathematical problems, because the SR value was 0, and the equation of this index (7) contains a division with natural logarithm of SR, hence there was a decrease, and thus it is not recommended to use this endpoint for characterisation.

Simpson index, similarly to the Shannon indices, represented the negative effects of nZnO. The rare species are rather more weighted in the Shannon than the common species, so the Shannon index is strongly influenced by species richness. The Simpson index weights common species more than relatively rare species, so this index gives more weight to evenness [60]. In our research, there was no significant difference between the two indices.

According to our results, McIntosh index is the best way to represent the change of the AWCD, AUC and SR endpoints; hence, the calculation of this index was based on the measured OD values, just like AWCD, AUC and SR. The McIntosh index is another measure of dominance; however, this index is not widely used. In our study, the McIntosh index value correlated with the microbial activity, and it also indicated the effect of treatment and time. Dejong [61] also found that the McIntosh and Simpson indices were more suitable to measure diversity than the Shannon index, because these indices were influenced noticeably more by evenness and less by richness than the Shannon index.

It should be highlighted that due to the sensitivity of the Gini coefficient, it reflected well the changes in metabolic activity triggered by the presence of NPs (nano titanium dioxide in addition to zinc oxide) (Figure S3). Gini index values increased with incremental ZnO NP concentrations, although the RMANOVA analysis did not display such a strong significant difference than in the case of the Shannon, Simpson and McIntosh index.

The Biolog EcoPlate™ technique does, however, have some limitations [32,49]. Using Biolog EcoPlate™ for bacterial community-level physiological profiling (CLPP) is easier and cheaper than DNA or RNA-based profiling. Additionally, several studies have reported that molecular profiling surveys are not more sensitive than CLPP approaches [62,63]. Nevertheless, further research is needed to estimate which bacterial genera are the most affected by NPs, because significantly different patterns can be observed by CLPP and DNA-based methods [64,65]. Changes of the aerobic and anaerobic microbial community should also be investigated, which could be performed by Biolog AN (anaerobic) microplate, provided with 95 carbon sources; hence, the use of more substrates could be examined.

## 5. Conclusions

Metal oxide nanoparticles are commonly and extensively used; therefore, they are likely to end up in aquatic environments. Nonetheless, their effect on freshwater microbial diversity has been poorly described. In this research, the Biolog EcoPlate™ technique was applied, based on living microorganisms, to show the metabolic capabilities and the physiological profiles of microbial community in MONPs affected freshwater. By experimenting with TiO_2_ and ZnO NPs in freshwater lab-scale microcosms, it was shown that nTiO_2_ weakly influences microbial community, while ZnO nanoparticles have a strong effect on the metabolic activity and functional diversity of the microbiome. Not just the presence and concentration of MONPs, but the exposure time also exhibited a significant effect on the studied Biolog EcoPlate™ parameters. The Biolog EcoPlate™ data clearly reflected that the changes were largely determined by the type of NP. Carbohydrates, carboxylic acids and polymers were found to be the principal indicator substrates at higher ZnO nanoparticle concentrations. Our results also demonstrated that the sensitivities of the examined indices were different and their simultaneous use could provide complementary information. McIntosh index as a diversity indicator displayed a good sensitivity for indicating the modified diversity of a bacterial community in nanomaterials affected freshwater. The evenness of microbial species and their carbon source use evenness also changed upon NP treatment, which was indicated by the Gini coefficient and Shannon evenness. To indicate the effect of nanoparticles on microbial communities the application of the Biolog EcoPlate™ as a rapid, cheap and practical technique is recommended.

## Figures and Tables

**Table 1:** Characteristics of freshwater samples from Lake Balaton.

Samples from Lake Balaton	pH ^(1)^	EC ^(2)^	NO_3_ (NO_2_)-N ^(3)^	K^+ (4)^	Na^+ (4)^	Ca^2+ (4)^	Mg^2+ (4)^	Cl^- (5)^	CO_3_^2- (6)^	HCO_3_^- (6)^	SO_4_^2- (7)^
	-	µS/cm	mg/L	mg/L	mg/L	mg/L	mg/L	mg/L	mg/L	mg/L	mg/L
Sample collected in July	8.3 ±0.2	707 ±16	0.22 ±0.10	6.7 ±0.2	36.4 ±0.4	33.9 ±	58.8 ±0.1	38.4 ±2.6	27.9 ±13.2	233.4 ±26.8	134.2 ±0.2
Sample collected in October	8.8 ±0.0	664 ±20	0.14 ±0.00	7.2 ±0.6	37.4 ±1.3	27.6 ±2.7	60.3 ±2.1	40.3 ±0.0	46.5 ±4.4	211.3 ±40.1	141.5 ±4.6

^(1)^ Determined by HS 21470-2:981; ^(2)^ Determined by HS EN 27888:1988; ^(3)^ Determined by ISO 7890-3:1988; ^(4)^ Determined by ICP-OES, HS EN 11885:2005; ^(5)^ Determined by EPA 9253:1994; ^(6)^ Determined by HS EN ISO 9963-1:1998; ^(7)^ Determined by HS 448-13:1983.

**Table 2:** RMANOVA results over time to evaluate effects of nZnO treatment on the freshwater microbial diversity. Bold numbers indicate significant differences at *p* < 0.05.

Source of Variation	d.f.	Mean Square	F Ratio	*p*-Value	Source of Variation	d.f.	Mean Square	F Ratio	*p*-Value
AWCD					SR				
Treatment	4	0.55	3317.68	0.000	Treatment	4	536.02	989.57	0.000
Time	2	0.01	7.95	0.041	Time	2	9.95	23.88	0.000
Time × Treatment	8	0.00	1.96	0.269	Time × Treatment	8	3.14	7.52	0.000
AUC					H				
Treatment	4	3.16	3299.60	0.001	Treatment	4	1.22	1487.4	0.000
Time	2	0.15	117.61	0.000	Time	2	0.18	25.2	0.000
Time × Treatment	8	0.03	19.46	0.006	Time × Treatment	8	0.10	13.5	0.000
Source of variation	d.f.	Mean square	F ratio	*p*-value	Source of variation	d.f.	Mean square	F ratio	*p*-value
E					U				
Treatment	3	3.38	223.07	0.000	Treatment	4	31.38	1230.25	0.000
Time	2	0.04	0.69	0.526	Time	2	0.70	0.70	0.000
Time × Treatment	6	0.05	0.78	0.606	Time × Treatment	8	0.23	0.23	0.000
D					G				
Treatment	4	0.02	50.87	0.000	Treatment	4	4.29	287.48	0.000
Time	2	0.01	30.71	0.000	Time	2	0.04	1.24	0.339
Time × Treatment	8	0.00	11.49	0.000	Time × Treatment	8	0.24	7.31	0.005

AWCD—the average well colour development, AUC—the area under the curve, SR—the substrate richness, H—Shannon diversity index, E—Shannon evenness index, D—Simpson index, U—McIntosh index, G—Gini coefficient.

**Table 3:** RMANOVA results over time to evaluate effects of nTiO_2_ treatment on the freshwater microbial diversity. Bold numbers indicate significant differences at *p* < 0.05.

Source of Variation	d.f.	Mean Square	F Ratio	*p*-Value	Source of Variation	d.f.	Mean Square	F Ratio	*p*-Value
AWCD					SR				
Treatment	4	0.01	7.01	0.028	Treatment	4	5.67	4.166	0.059
Time	2	0.04	52.87	0.000	Time	2	21.30	26.438	0.000
Time × Treatment	8	0.00	4.49	0.015	Time × Treatment	8	5.14	6.381	0.002
AUC					H				
Treatment	4	0.25	35.38	0.000	Treatment	4	0.01	6.4	0.034
Time	2	0.24	53.20	0.000	Time	2	0.05	40.9	0.000
Time × Treatment	8	0.04	9.60	0.000	Time × Treatment	8	0.00	2.1	0.132
Source of variation	d.f.	Mean square	F ratio	*p*-value	Source of variation	d.f.	Mean square	F ratio	*p*-value
E					U				
Treatment	4	0.00	5.4	0.046	Treatment	4	0.34	6.80	0.030
Time	2	0.00	2.7	0.114	Time	2	1.26	107.30	0.000
Time × Treatment	8	0.00	4.3	0.018	Time × Treatment	8	0.10	8.75	0.001
D					G				
Treatment	4	0.00	2	0.239	Treatment	4	0.01	7.426	0.025
Time	2	0.00	46	0.000	Time	2	0.03	122.266	0.000
Time × Treatment	8	0.00	2	0.103	Time × Treatment	8	0.00	4.887	0.011

AWCD—the average well colour development, AUC—the area under the curve, SR—the substrate richness, H—Shannon diversity index, E—Shannon evenness index, D—Simpson index, U—McIntosh index, G—Gini coefficient.

## Data Availability

Not applicable.

## Supplementary Materials

The following are available online at https://www.mdpi.com/article/10.3390/nano11071777/s1, Figure S1: The effect of TiO_2_ nanoparticles on freshwater based on the area under the curve value, Figure S2: The effect of TiO_2_ nanoparticles on Shannon evenness value of freshwater, Figure S3: The effect of TiO_2_ nanoparticles on freshwater Simpson index value, Figure S4: The effect of TiO_2_ nanoparticles on freshwater Gini index value, Table S1: Pearson correlation results to evaluate coherence of nZnO concentrations and diversity indices, Table S2. Pearson correlation results to evaluate coherence of nTiO_2_ concentrations and diversity indices.
