# Supplementary material for: The Biolog EcoPlate™ Technique for Assessing the Effect of Metal Oxide Nanoparticles on Freshwater Microbial Communities
Source: Nanomaterials (Basel). 2021 Jul 8;11(7):1777. doi: 10.3390/nano11071777 (PMC8308119; doi:10.3390/nano11071777)
Supplement: Supplementary file 1 [file nanomaterials-11-01777-s001.zip › nanomaterials-1257607-supplementary.pdf]

Table S1. Pearson correlation results to evaluate coherence of nZnO concentrations and diversity indices. Bold numbers indicate significant differences at p<0.05.

	r											
	AWCD			AUC			SR			H		
	3rd day	7th day	14th day	3rd day	7th day	14th day	3rd day	7th day	14th day	3rd day	7th day	14th day
Concentration	**-0.997**	**-0.999**	**-0.989**	**-0.999**	**-1.000**	**-0.985**	**-0.993**	**-0.999**	**-0.972**	**-0.997**	-0.971	**-0.990**

	r											
	E			D			U			G		
	3rd day	7th day	14th day	3rd day	7th day	14th day	3rd day	7th day	14th day	3rd day	7th day	14th day
Concentration	-0.600	**-0.723**	**-0.614**	**-0.641**	**-0.887**	**-0.849**	**-0.835**	**-0.838**	**-0.808**	**0.894**	**0.824**	**0.903**

AWCD—the average well colour development, AUC—the area under the curve, SR—the substrate richness, H—Shannon diversity index, E—Shannon evenness index, D—Simpson index, U—McIntosh index, G—Gini coefficient

In general, the area under the curve (AUC) results demonstrated similar patterns of the nTiO_2-induced effects on the microbial community than the AWCD (Figure S1), but the adverse effects on AUC were higher than on AWCD. The AUC values usually showed a decreasing tendency with incremental ENP concentrations in the microcosms. The highest decrease (30%) occurred at 100 mg/L nTiO_2 concentration after 3 days. The extent of the effects decreased with the increase of exposure duration. According to the correlation analysis (Table S2.) the AWCD values negatively correlated (r=-0.748) with the nTiO_2 concentration after 2 weeks exposure at p<0.05. RMANOVA analysis indicated that treatment, time and their interaction had significant effect on the AUC values (Table 3).

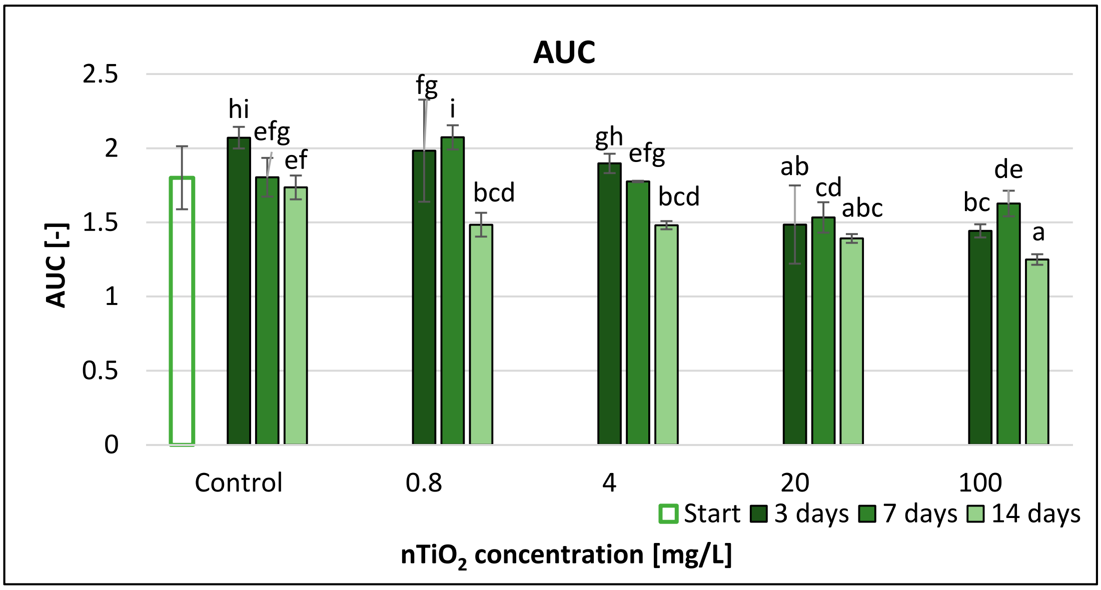

Figure S1. The effect of TiO_2 nanoparticles on freshwater based on the area under the curve value. Letters on the columns indicate significant differences (level of significance: p < 0.05).

Shannon evenness (E), derived from the Shannon index (H), exhibited slight changes (2–7%) but the effects were significant compared to the control at all the tested TiO_2 concentrations after 3 and 7

days (Figure S2). Nevertheless, the values in neither case presented a linear association between the concentration and Shannon index (Table S2).

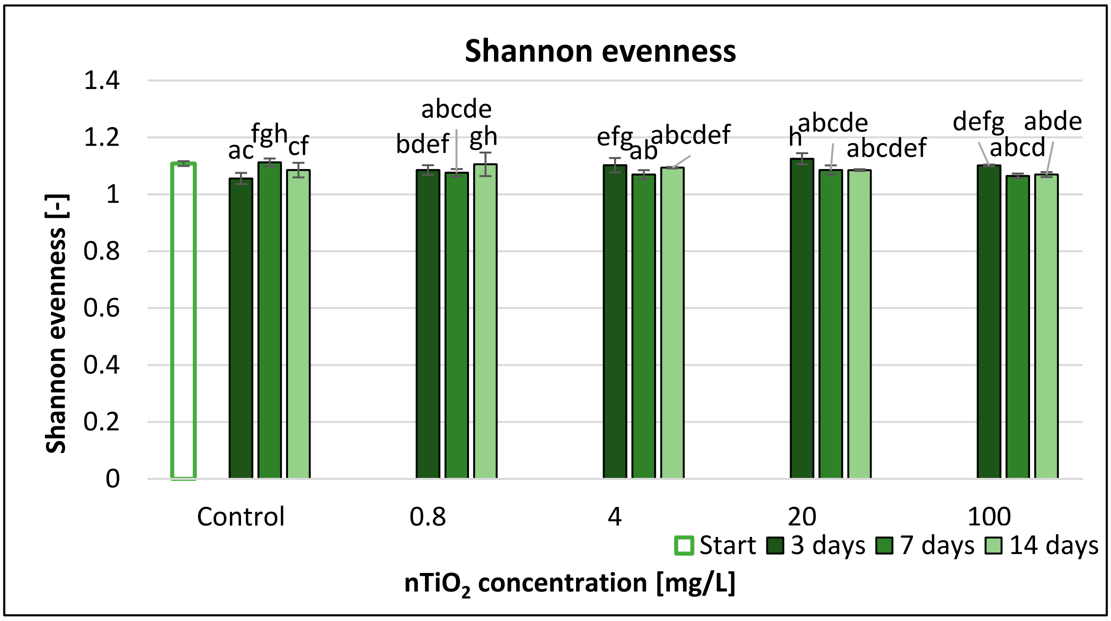

Figure S2. The effect of TiO_2 nanoparticles on Shannon evenness value of freshwater. Letters on the columns indicate significant differences (level of significance: $p < 0.05$).

The Shannon evenness showed a significant difference from the control at 0.8 and 100 mg/L $nTiO_2$ concentrations after 14 days, and the correlation coefficients demonstrated medium association (negative correlation, r=-0.647) in this case (Table S2). Examining the effects of the factors by RMANOVA (Table 3), it can be seen that exposure time had significant effect on the evolution of Shannon evenness; the effect of treatments was different at different sampling times. Additionally, the time interacted with the effect of treatments.

The sensitivity of the Simpson index was lower than that of the Shannon index (Figure S3). Simpson indices exhibited minor changes in the presence of TiO_2 NPs in the microcosms. The value of the Simpson index was maintained at around 0.94–0.95 during the experiment. This endpoint was significantly different from the control only in one case, at 100 mg/L $nTiO_2$ after 3 days. Regarding the effect of $nTiO_2$ concentration, the results of RMANOVA analysis supported our observations, demonstrating that treatments did not influence significantly the uniformity of microbial community (Table 3). Nevertheless, the effect of treatments differed significantly in time. Correlation analysis (Table S2) indicated medium non-significant association between TiO_2 NP concentration and Simpson index.

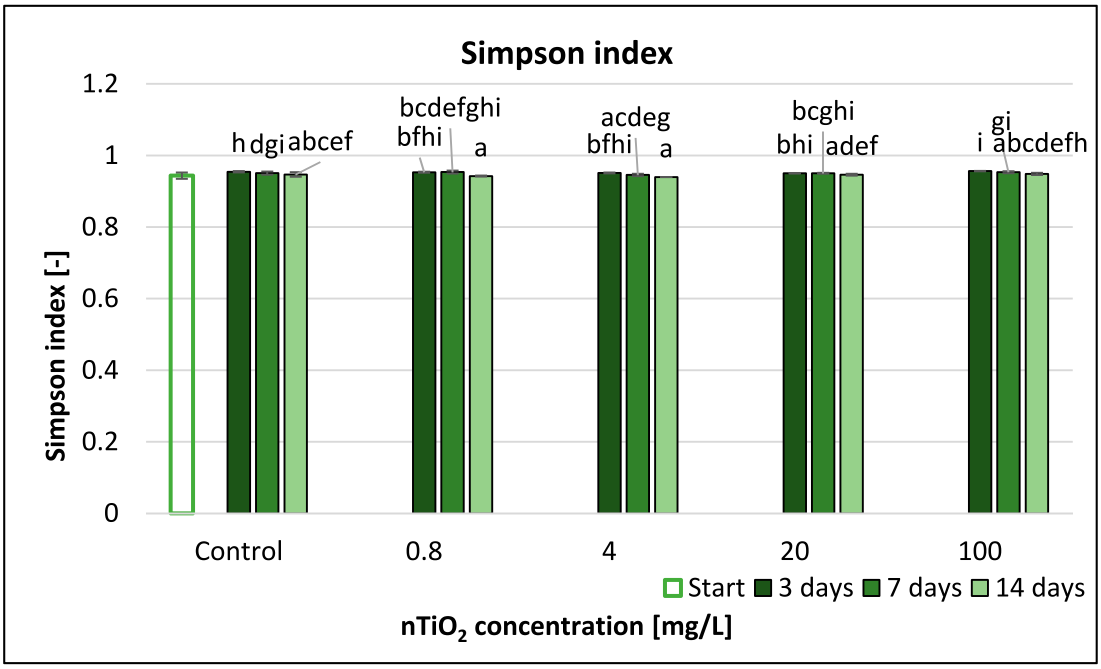

Figure S3. The effect of TiO_2 nanoparticles on freshwater Simpson index value. Letters on the columns indicate significant differences (level of significance: $p < 0.05$).

The value of the Gini index increased over time for all treatments, while the $nTiO_2$-induced effect on the Gini index differed at each time point (Figure S4). There was a significant difference compared to control only after 7 days at 100 mg/L $nTiO_2$ concentration, resulting in an 11% decrease in the Gini index. Correlation analysis (Table S2) indicated a medium non-significant linear association between TiO_2 NP concentration and Gini index. The RMANOVA analysis of the Gini index results illustrated clear significant effect of treatment and time, as well as of their interaction (Table 3.).

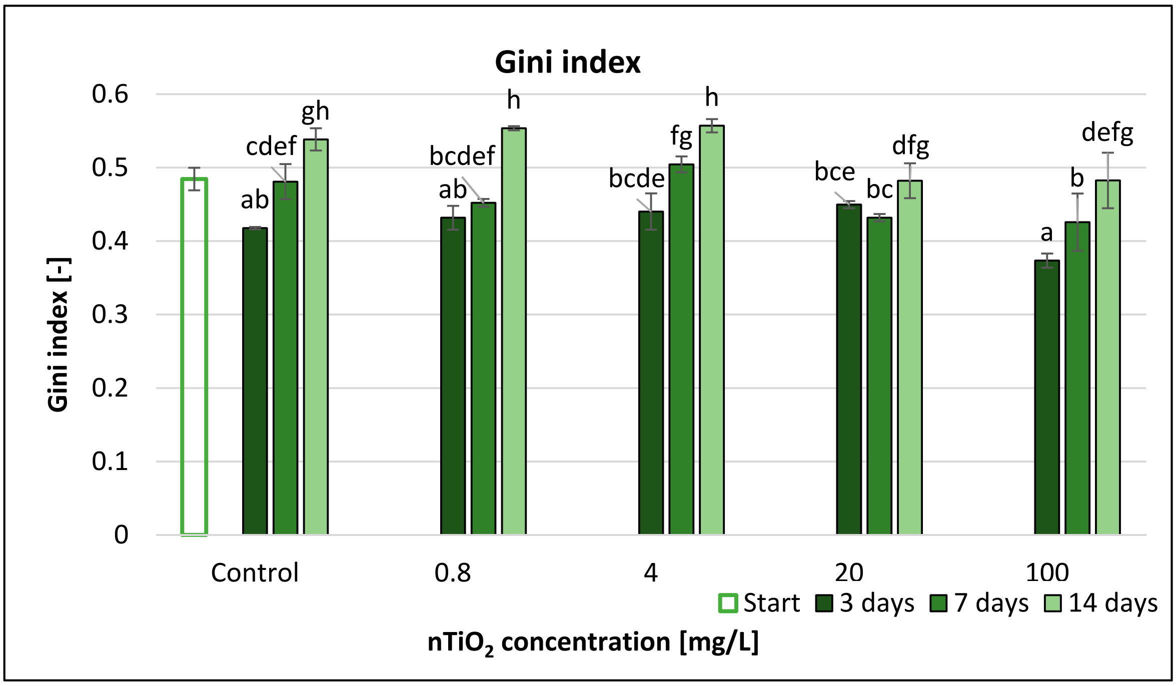

Figure S4. The effect of TiO_2 nanoparticles on freshwater Gini index value. Letters on the columns indicate significant differences (level of significance: $p < 0.05$).

Table S2. Pearson correlation results to evaluate coherence of $nTiO_2$ concentrations and diversity indices. Bold numbers indicate significant correlations at *p*<0.05.

	r											
	AWCD			AUC			SR			H		
	3rd day	7th day	14th day	3rd day	7th day	14th day	3rd day	7th day	14th day	3rd day	7th day	14th day
Concentration	-0.193	-0.380	-0.364	-0.614	-0.479	**-0.748**	-0.011	0.451	**0.850**	0.803	0.188	0.582
	r											
	E			D			U			G		
	3rd day	7th day	14th day	3rd day	7th day	14th day	3rd day	7th day	14th day	3rd day	7th day	14th day
Concentration	0.226	-0.379	**-0.647**	0.538	0.434	0.377	-0.425	-0.529	-0.474	-0.809	-0.676	-0.665

AWCD – the average well colour development, AUC—the area under the curve, SR—the substrate richness, H—Shannon diversity index, E—Shannon evenness index, D—Simpson index, U—McIntosh index, G—Gini coefficient
